# Age-related loss of mitochondrial glutathione exacerbates menadione-induced inhibition of Complex I

**DOI:** 10.1016/j.redox.2019.101155

**Published:** 2019-03-02

**Authors:** Nicholas O. Thomas, Kate P. Shay, Tory M. Hagen

**Affiliations:** a Linus Pauling Institute, Oregon State University, Corvallis, OR, 97331-6512, USA; b Department of Biochemistry and Biophysics, Oregon State University, Corvallis, OR, 97331-7305, USA

**Keywords:** Aging, Redox cyclers, Mitochondria, Glutathione, Respiratory reserve capacity

## Abstract

The role of mitochondrial GSH (mGSH) in the enhanced age-related susceptibility to xenobiotic toxicity is not well defined. We determined mGSH status and indices of mitochondrial bioenergetics in hepatocytes from young and old F344 rats treated with 300 μM menadione, a concentration that causes 50% cell death in old. At this concentration, mGSH was significantly lost only in hepatocytes from old rats, and with near total depletion due to lower basal mGSH in aged cells. In old hepatocytes, menadione caused mitochondrial membrane potential to collapse, as well as significant deficits in maximal O_2_ consumption and respiratory reserve capacity, indicators of cellular bioenergetic resiliency. Further examination revealed that the menadione-mediated loss of respiratory reserve capacity in aged hepatocytes was from significant inhibition of Complex I activity and increased proton leak, for which an increase in Complex II activity was not able to compensate. These data demonstrate an age-related increase in mitochondrial susceptibility to a redox-cycling challenge, particularly in regards to Complex I activity, and provide a plausible mechanism to link this vulnerability to mGSH perturbations.

## Graphical abstract

**Figure:**
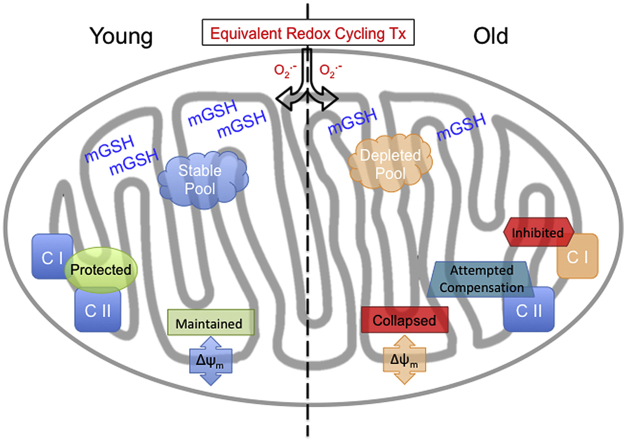

## 1 Introduction

Aging is characterized by a decline in cellular redox homeostasis and detoxification capacity, which is coincident with an increased risk for age-related pathophysiologies [[1], [2], [3], [4], [5], [6], [7], [8], [9]]. Mitochondrial dysfunction and decay is an underlying factor that has been implicated in aging and age-related diseases [10]. Characteristics of this decay include decreased mitochondrial membrane potential (Δψ_m_), basal respiration rate, and respiratory reserve capacity (RRC), a measure of mitochondrial elasticity to respond to energy requirements. Mitochondria from aged tissues display increases in oxidant leakage, mitochondrial DNA damage, and susceptibility to the formation of the mitochondrial permeability transition pore (MPTP), leading to apoptotic initiation [[11], [12], [13]]. Mitochondrial matrix glutathione (mGSH) is a critical regulator of mitochondrial ATP production in that it modulates critical protein sulfhydryl redox states, which in turn influences both NADH and FADH_2_ generation and electron flow through the electron transport chain (ETC) [14,15]. Complex I in particular has critical, redox active thiols that can be reversibly glutathionylated in order to regulate electron flux during increased oxidative stress [[16], [17], [18], [19]]. Finally, GSH constitutes one of the primary defenses against oxidative damage both as an antioxidant and as a substrate for multiple detoxification enzymes such as the glutathione-S-transferases (GSTs), glutaredoxins (Grxs), and glutathione peroxidases (GPxs). Thus, mGSH is critical to maintaining mitochondrial function and as such, significantly influences cell and tissue survival. In fact, studies done by Fernandez-Checa, Garcia-Ruiz, and others [12,13,20,21] have shown that while cells can survive near-total loss of the cytosolic GSH fraction, when mGSH is compromised to even a small degree, Δψ_m_ collapses, cells become sensitized to oxidative insult, and intrinsic apoptotic and necrotic pathways are instigated. Thus, the mGSH pool constitutes a unique subcellular fraction that must be maintained in a narrowly defined equilibrium for proper mitochondrial and cellular function.

In spite of the important linkage between maintenance of mGSH and normal cell metabolism, our lab and others have shown that mGSH decreases by as much as 50% with age [[22], [23], [24]]. While the age-related loss of mGSH may be attributable to a variety of factors such as changes in membrane fluidity, decreased transport, or greater GSH utilization due to an increasingly oxidant-rich environment, our lab showed that hepatocellular GSH synthesis declines with age, which may also significantly alter mGSH levels [4]. In this regard, we showed that declines in Nrf2-dependent expression of GSH synthesis enzymes limited the basal GSH synthesis capacity and sensitized hepatocytes from old rats to acute administration of menadione, a redox cycling agent [25]. Specifically, decreased basal levels of GSH and the GSH-dependent lipid peroxidase, GPx4, led to an increased rate and magnitude of lipid peroxidation and a concomitant loss in cell viability. Furthermore, the importance of GSH-dependent detoxification was demonstrated by the ability to remediate this increased vulnerability by providing a substrate for GSH synthesis (N-acetyl-l-cysteine). While our prior work demonstrated that increased hepatocellular susceptibility to menadione was mediated through age-related losses of GSH *per se*, it did not investigate the role of the distinct mGSH pool in contributing to the hepatic vulnerability to redox cycling agents. To fill this important gap in knowledge, the objective of the present work is to determine whether age-related deficits in mGSH specifically contribute to vulnerability to menadione-mediated insults. Based on this objective, we hypothesized that age-related increases in vulnerability to an acute redox cycling challenge are due to a failure of mGSH capacity to maintain mitochondrial ETC function.

In order to test this hypothesis, we utilized an acute menadione dose (300 μM) in hepatocytes isolated from young and old male Fischer 344 rats (F344) and evaluated age-related differences in measures of mitochondrial function. In addition to the aforementioned increased vulnerability with age, other studies show that menadione directly interacts with Complex I in the mitochondria, depletes mGSH, decreases Δψ_m_, and induces formation of the MPTP [[26], [27], [28]]. As such, menadione is an appropriate model toxin to explore the role of age-related decline in mGSH and loss of mitochondrial function in reduced capacity to respond to acute exposure to redox cycling agents. Herein, we show that age-related decline in mGSH increases mitochondrial vulnerability to acute menadione exposure via loss of ETC Complex I activity, a concomitant loss in basal O_2_ consumption and RRC, and finally, collapse of Δψ_m_.

## 2 Results

### 2.1 Acute menadione administration causes rapid mitochondrial glutathione loss

**Fig. 1:**
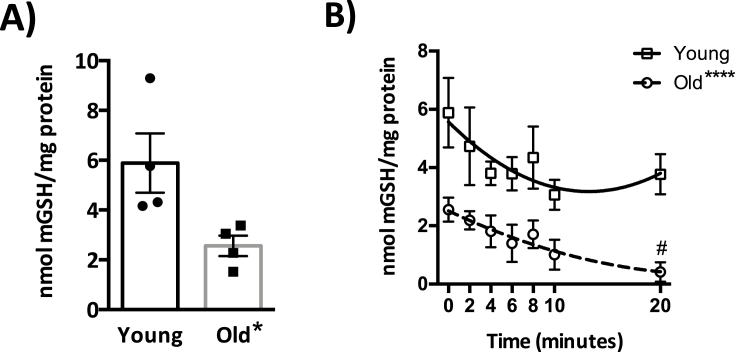
**An age-related mitochondrial glutathione (mGSH) deficiency is evident both before and during redox stress**. A. Basal mGSH level in old rat hepatocytes versus those of young (Student's t-test, *p = 0.0388, N = 4). B. Effects of age on mGSH loss and following a 300 μM menadione treatment. Two-way ANOVA demonstrated no significant interaction between age and time-course (p = 0.9358), but there were differences between age groups (*p < 0.0001, N = 4) and over the time-course within age groups (p = 0.05, N = 4).

In a prior study, we established that hepatocytes from old rats were nearly 50% more susceptible to menadione (LC_50_ young: 405 μM; old: 275 μM) with significant differences in GSH loss and increased markers of lipid peroxidation within minutes [25]. To determine whether the enhanced toxicity from menadione insult was mediated at least in part through mitochondrial dysfunction, we treated hepatocytes isolated from young and old F344 rats with 300 μM menadione (the ∼LC_50_ for old) and assayed mGSH levels over a 20 min time-course. Basal mGSH level (Fig. 1A) in old rat hepatocytes was 43% of that found in the young, and while menadione treatment caused a continuous decline in mGSH levels in old that became significant by the end of the time-course, there was only a trend of decline and recovery in young with no significant loss of mGSH (Fig. 1B). Moreover, mGSH levels in young remained significantly higher versus old cells throughout the menadione exposure (Fig. 1B). These results suggest that menadione causes marked losses in mGSH regardless of age, but mitochondria from old rat hepatocytes are less resilient owing to significantly lower steady-state mGSH levels prior to menadione additions, and the lack of recovery following menadione treatment. In addition to mGSH measures, we attempted to monitor the mitochondrial GSH:GSSG ratio. However, the GSSG quantification was below the signal to noise ratio, preventing this measure from being obtained (data not shown).

### 2.2 Δψ_m_ collapses under an acute menadione challenge

**Fig. 2:**
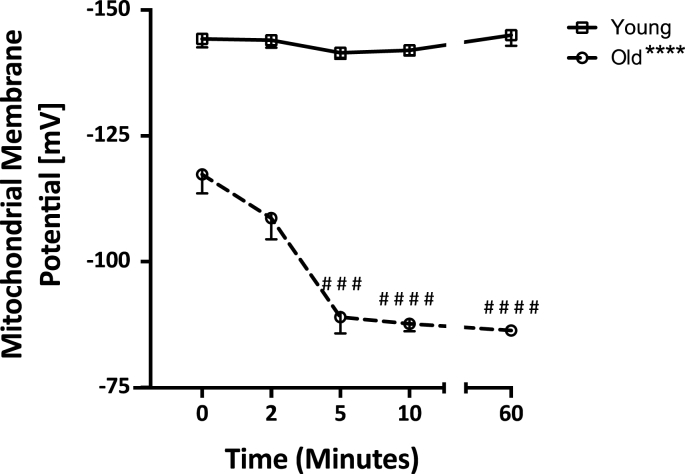
**Challenge with menadione (300 μM), results in the collapse of mitochondrial membrane potential (Δψ**_**m**_**) only in old rat hepatocytes**. Menadione effects on Δψ_m_, as measured by a TPP-electrode, were determined in hepatocytes isolated from young and old rats. Two-way ANOVA demonstrated a significant interaction between the age and changes in GSH over time following menadione exposure (p < 0.0001, N = 4). Simple main effects analysis showed significant effects between age groups (****p < 0.0001, N = 4) as well as over the time-course (versus t_0_) in old rat hepatocytes from 5 min after menadione onwards (###p < 0.001, ####p < 0.0001, N = 4). There were no significant changes in Δψ_m_ hepatocytes from young rats following menadione administration (p > 0.05, N = 4).

Because previous studies have shown that mGSH depletion adversely affects mitochondrial bioenergetics and initiates cell death [[29], [30], [31], [32]], we asked the question whether the menadione-induced loss of mGSH in the aged rat hepatocytes was sufficient to adversely affect mitochondrial function. We initially examined Δψ_m_ as a relevant parameter of overall mitochondrial function because it is the major component of the proton motive force, the electrochemical gradient utilized by mitochondria to perform ATP synthesis, in rat liver [33,34]. By measuring Δψ_m_ using a tetraphenylphosphonium (TPP) electrode, we showed that there was an 18.7% age-related loss of baseline Δψ_m_ (26.9 ± 3.1 mV) in old versus young cells, indicating that losses in this key driving force for ATP synthesis correlated both with mGSH deficits and age. Menadione administration caused a collapse in Δψ_m_ in old but not young rat hepatocytes. As shown in Fig. 2, Δψ_m_ in old rat hepatocytes was 24.1% lower than baseline following menadione addition (−28.3 ± 1.5 mV), but cells from young animals showed no appreciable deficits relative to controls. In fact, the menadione-mediated decline in Δψ_m_ in aged rat hepatocytes breached the threshold known to induce mitochondrial permeability transition pore opening (∼−100 mV) [[35], [36], [37], [38]].

Further analysis revealed that Δψ_m_ in young rat hepatocytes that had been treated with menadione remained at initial values approximately 1 h after treatment; however, no recovery in Δψ_m_ was evident in old rat hepatocytes. These results demonstrate a striking age-associated sensitivity of Δψ_m_ to menadione in both the magnitude of membrane potential decline and in its limited recovery potential with age.

### 2.3 Mitochondrial basal respiration and respiratory reserve capacity are attenuated under menadione challenge

**Fig. 3:**
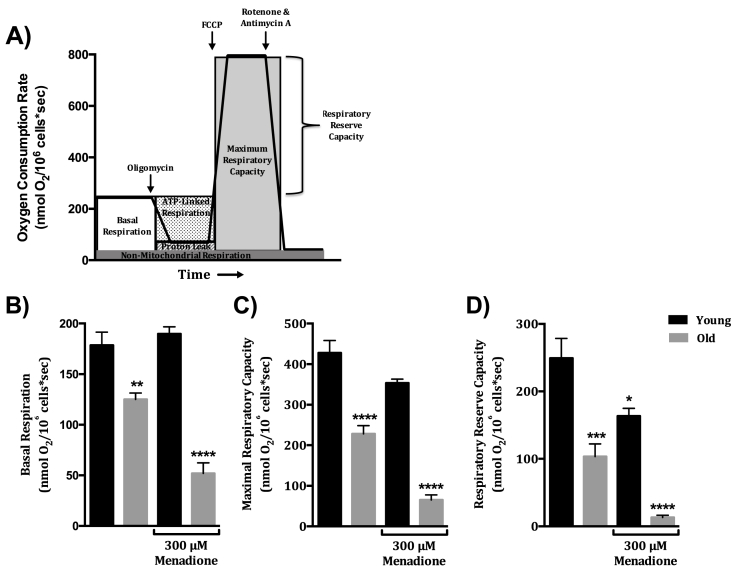
**Menadione-induced loss of respiratory reserve capacity (RRC) is exacerbated with age**. A. Representative O_2_ consumption profile of hepatocytes taken from a young rat highlighting basal and maximal O_2_ consumption states as well as the RRC. B. Basal O_2_ consumption with age is significantly reduced both without and with 300 μM menadione (**p < 0.01, ****p < 0.0001, N = 4). C. Maximal respiratory capacity with age is significantly reduced both without and with 300 μM menadione (****p < 0.0001, N = 4), while young rat hepatocytes are not significantly affected (p = 0.059, N = 4). D. RRC is significantly decreased with age (***p < 0.001, N = 4) and in hepatocytes treated with 300 μM menadione from both young (*p < 0.0314, N = 4) and old (****p < 0.0001, N = 4) rats. Data shown as mean ± SEM of N = 4 experiments, statistical significance determined by one-way ANOVA with Sidak multiple comparisons post-test.

Having established that age exacerbates menadione's effects on Δψ_m_, we hypothesized that this should translate to decrements in mitochondrial O_2_ consumption characteristics. An examination of O_2_ consumption characteristics in young and old rat hepatocytes in the presence and absence of various substrates and with or without menadione addition was undertaken (Fig. 3A). Results showed that basal O_2_ consumption was detrimentally affected in hepatocytes from old rats and this deficit was significantly exaggerated when menadione was given (Fig. 3B: 34% and 71% respectively).

To better define whether menadione's effect on mitochondrial O_2_ consumption was from inhibiting electron transport function, uncoupled maximal O_2_ consumption rate (maximal OCR) was monitored in digitonin-permeabilized hepatocytes following additions of carbonyl cyanide 4-(trifluoromethoxy)phenylhydrazone (FCCP), a protonophore. Consistent with results shown in Fig. 3A, maximal OCR in aged hepatocytes was significantly reduced both without and with 300 μM menadione treatment (47% and 85% losses respectively versus young controls, Fig. 3C). Maximal OCR in young rat hepatocytes was not significantly affected by menadione at the concentration given (p = 0.059). Additionally, we calculated RRC, which is the difference between the FCCP-mediated maximal OCR and basal OCR, and is a measure of mitochondrial elasticity to respond to energy requirements. RRC declined dramatically with age and when dosed with menadione in old rat hepatocytes (59% and 95% loss respectively versus young controls, Fig. 3D) while hepatocytes from young rats were not as affected. Based on these results, we conclude that mitochondria lose the capacity to respond to a menadione exposure with age, which leads to a rapid deficit in cell bioenergetics.

### 2.4 Acute menadione exposure significantly disrupts ETC Complex I in old rat hepatocytes

**Fig. 4:**
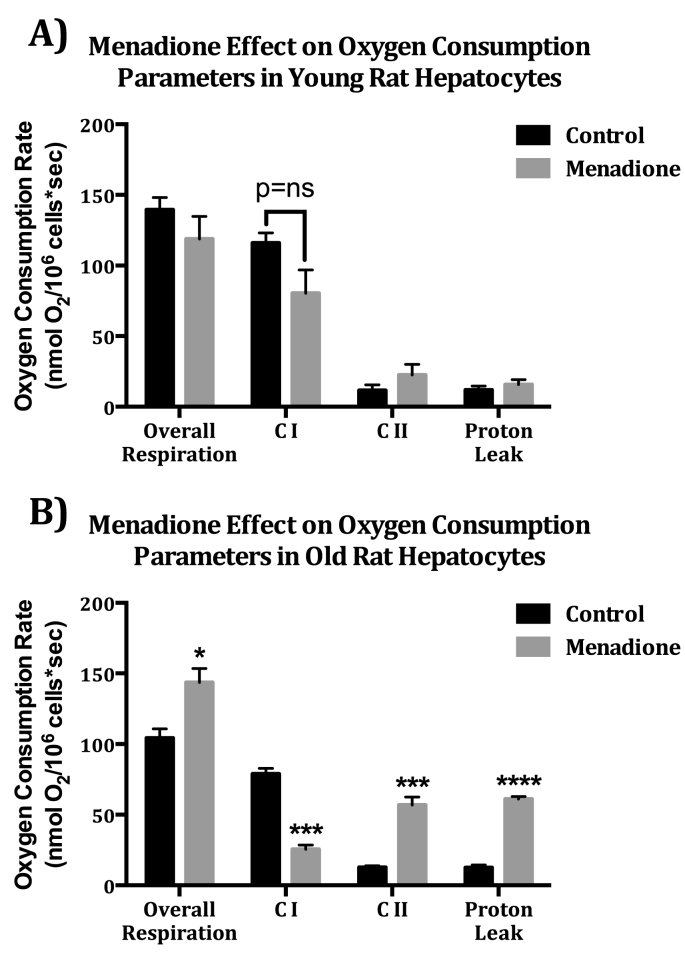
**Menadione treatment (300 μM) causes marked age-related differences in O**_**2**_**consumption parameters** A. In young rat hepatocytes, menadione exposure did not have a significant effect on overall O_2_ consumption, or O_2_ consumption linked to Proton Leak, CI, or CII (Student's t-test, N = 4) B. In hepatocytes from old rats, overall O_2_ consumption as well as O_2_ consumption linked to Proton Leak, CI, and CII were significantly different. (*p = 0.03105, ***p < 0.001, ****p < 0.0001, N = 4, Student's t-test).

In order to correlate the age-dependent loss in mitochondrial resiliency to menadione administration with the severe declines in mGSH also evident with menadione, we monitored the activities of ETC components in young and old rat hepatocytes with and without menadione. This analysis was undertaken because of the acknowledged sensitivity of Complex I sulfhydryls to menadione. Hepatocytes from young and old rats were treated with 300 μM menadione for 15 min and mitochondrial O_2_ consumption was assessed. Complex I-linked (CI), and Complex II-linked (CII) O_2_ consumption were examined in the presence of glutamate and succinate (CI and CII substrates). Further differentiation of CI and CII O_2_ consumption was ascertained by addition of rotenone, an inhibitor of Complex I in the ETC. Following assessment of basal O_2_ consumption, proton leak-linked O_2_ consumption (Leak) was measured after injection of oligomycin, an ATP synthase inhibitor that blocks the F_O_ subunit of the proton channel. “Leak” is thus defined as proton leak, proton slip, cation cycling, and electron leak and their contribution to a reduction in biochemical coupling efficiency and the dissipation of the proton gradient that is not available for ATP production [39]. In young rat hepatocytes, menadione treatment did not have a significant effect on overall O_2_ consumption or O_2_ consumption linked to CI, CII, or Leak (Fig. 4A). In hepatocytes from old rats, O_2_ consumption contributions for all of these measurements were significantly different. While CI O_2_ consumption decreased by 3.1-fold, overall O_2_ consumption was increased by 38% due to CII and Leak O_2_ consumption increasing by 4.5-fold and 4.9-fold respectively (Fig. 4B). These findings suggest that there is an age-related susceptibility to CI inhibition from redox cycling oxidative stress, and that there is a potentially compensatory, but insufficient increase in CII O_2_ consumption in conjunction with increased Leak which leads to an increase in overall, but not ATP-linked, O_2_ consumption.

## 3 Discussion

The aging process leads to declines in cellular function and resilience. The hepatic mitochondrial antioxidant system is in decline with GSH being one of the more prominent defense mechanisms adversely effected with age. Even though it is plausible that our findings are glutathione related, we cannot rule out other mitochondrial antioxidant enzymes (e.g. Grx2, GPx4, SOD) that decline with age [24,40], may be part of the mechanism of the accentuated menadione toxicity we have observed. Our data points to loss of GSH as a major factor in the age-related increase in vulnerability to redox cycling oxidative stressors. GSH is independently maintained in many cellular compartments and mGSH is not in equilibrium with GSH in other cellular compartments [41]. The nuclear GSH pool is also separately maintained [42,43]. However, it remains unknown if this fraction changes with age. While our fractionation method releases cytosolic GSH, it is unclear to what extent the nuclear GSH pool is released or contributes to our data shown in Fig. 1. However, our prior work demonstrated that this methodology results in collection of ∼15–20% of the total GSH pool which is in keeping with the known mGSH fraction. This suggests that the mGSH pool is likely what our present study is monitoring. We and others have observed that the age-related decline in mGSH is more severe than in the cytosol [44] and the endoplasmic reticulum [23]. Moreover, the age-related deficits in mGSH may have more detrimental consequences; while loss of cytosolic GSH can be compensated for with other antioxidants during an oxidative stress, mGSH is critical to normal mitochondrial function, which includes acting as a redox modulator of **ETC** proteins, an antioxidant, a detoxicant of electrophilic xenobiotics, a stabilizer of mitochondrial DNA, and as an essential cofactor for Fe-S cluster synthesis [[45], [46], [47], [48], [49]], and hence cellular survival [13]. In this work, we show the crucial interdependence between the loss of GSH-dependent measures of function and resilience to menadione challenge. Ideally, it would be salient to directly test the role of mGSH in this phenomenon by modeling the aging phenotype through specific depletion of mGSH levels in young rat hepatocytes to that evident in old. However, we have found no commercially available agent that does not also significantly alter cytosolic GSH in the timeframe of our experiments. Thus, the role of mGSH in the age-related susceptibility of mitochondria to menadione and perhaps other redox cycling agents, remains to be fully elucidated.

The current study focused on susceptibility to mitochondrial dysfunction during acute menadione insult in freshly isolated intact and digitonin-permeabilized hepatocytes from young and old rats. This cell model is appropriate for understanding aging differences in mitochondrial dysfunction because F344 rat hepatocytes reflect the age-related losses of GSH, mGSH, and mitochondrial function that are seen in humans [[50], [51], [52]]. The use of permeabilized cells offers the advantages of both isolated mitochondria and intact cells without suffering from the disadvantages inherent to each of those methods. For example, digitonin-permeabilized cells maintain the mitochondrial network structure, as well as cellular compartment interactions seen in intact cell models [35]. Additionally, it allows for the analysis of mitochondria function within minutes of treating cells and maintains Δψ_m_, which limits mGSH redistribution which would normally occur when isolating mitochondria by standard differential centrifugation techniques [53]. It also offers the direct control of ETC substrate composition and concentrations like in isolated mitochondria, but avoids mitochondrial damage and inadvertent selection for the least fragile mitochondria that often occur during the isolation [35].

We used menadione in the current study because the mechanisms of its toxicity and detoxification have been thoroughly explored. Menadione is a potent redox cycling compound that generates a strong, persistent oxidative stress via the initial generation of superoxide followed by its dismutation into more deleterious ROS/RNS [26,54]. We have previously demonstrated that the age-related increase in vulnerability to acute challenge with this compound is GSH-dependent [25]. Additionally, menadione treatment in isolated mitochondria was previously demonstrated to deplete GSH and inhibit Complex I activity [27,55]. However, prior to this report, we are not aware of any studies showing susceptibility of mitochondria to menadione as a function of age in hepatocytes. This distinction is important because it allows us to differentiate age-related effects of detoxification mechanisms in the cytosolic and mitochondrial compartments. For example, NqO1, the principal enzyme that detoxifies the menadione parent compound, is almost entirely localized to the cytosol, and has been shown to have 4- and 9-fold higher protein and activity levels in aged rat hepatocytes [25,56]. In contrast, GPx4 is largely found in mitochondria, and its protein levels decline by 70% in aged rat hepatocytes [[57], [58], [59]]. Thus, treatment of intact cells followed by rapid permeabilization and analysis of mitochondrial function gives a better understanding as to the relative importance of cytosolic versus mitochondrial toxicity and detoxification changes with age.

Loss of mGSH happens almost immediately after being dosed with menadione in both young and old hepatocytes. However, menadione-induced mGSH loss in cells from young animals was relatively limited, did not rise to statistical significance and remained above the steady-state basal amounts of mGSH found in old rats. This is a critical observation because the rapid mGSH loss in aged rat cells precedes severe declines in Δψ_m_, loss of RRC, and the inhibition of Complex I electron flux. This suggests that the lower basal level of mGSH plays a critical role in the age-related loss of detoxification resiliency under an acute redox cycling exposure. While a causal relationship was not determined in this work, mGSH involvement in each of these markers of mitochondrial function is well established [13,16,35].

Our results further demonstrate that age increases mitochondrial susceptibility to acute redox cycling challenge, and that Complex I in particular is more vulnerable to inhibition when exposed to a redox cycling agent. Previous studies have demonstrated an age-related shift toward a more oxidized mitochondrial environment [17,18]. This includes an increase in the GSSG content and concomitant glutathionylation of mitochondrial proteins. Reversible glutathionylation of critical cysteine residues on the 51 and 75 kDa subunits of Complex I have been shown to occur when mGSH is depleted during increased oxidizing conditions in the mitochondria [19,[60], [61], [62]]. Creation of this mixed disulfide could potentially account for the basal loss of Complex I activity, basal O_2_ consumption and Δψ_m_ with age [[63], [64], [65]]. Additionally, our lab showed that activity of glutaredoxin 2 (Grx2), the mitochondrial enzyme that removes glutathione from redox active protein sulfhydryls (like those on Complex I), decreases with age in rat cardiac mitochondria [24]. Other reports show a similar decline in Grx2 that cells from patients with Werner syndrome, a disease characterized by premature aging [66]. Combined with our previous work which demonstrated that GSH maintenance alone can remediate the loss of resilience seen with aging, and the established importance of mGSH regulation of Complex I activity, the evidence strongly suggests that age-related loss of mGSH plays a critical role in the demonstrated mitochondrial sensitization. Therefore, these results provide a plausible mechanism that links mGSH redox perturbations during aging and menadione insult, and the significant decline in Complex I activity.

Aside from exploring the mechanisms discussed above, this work identifies mGSH as a potential target for age-related therapeutics. Our previous work demonstrated that a two-week treatment with lipoic acid was capable of increasing both cytosolic and mGSH. However this approach is not directly applicable to the present acute exploration since lipoic acid upregulated Nrf2-dependent GSH synthesis genes over the course of days to weeks versus the strictly acute conditions applied herein. Nevertheless, lipoic acid as well as other compounds that induce GSH synthesis (e.g. n-acetyl cysteine) may be a long-term option for prophylactic therapies to prevent loss of mGSH with age in order to limit mitochondrial susceptibility to xenobiotics that may ultimately affect the organelle's function. Additionally, we are utilizing a novel compound (patent pending) to direct GSH to mitochondria, thus avoiding slow or limited uptake from the cytosol, which may be limited with age. Such strategies may act as an interventional means to protect mitochondria under acute electrophilic insult.

## 4 Methods and materials

### 4.1 Reagents

Mitochondrial respiration medium (MiR05) was prepared as described by Gnaiger et al. (see www.bioblast.at/index.php/MiPNet14.13_Medium-MiR06). In brief, MiR05 medium, pH 7.1, contains 0.5 mM EGTA, 3 mM MgCl_2_-6H_2_O, 60 mM lactobionic acid, 20 mM taurine, 10 mM KH_2_PO_4_, 20 mM HEPES, 110 mM d-Sucrose, and 1 g/L BSA (Fraction V, fatty acid free). Collagenase, type IV, was purchased from Worthington Biochemical Corporation (Lakewood, NJ). High purity digitonin, a non-ionic detergent that interacts specifically with cholesterol and permeabilizes cell membranes, was purchased from Millipore-Sigma (Cat# 300410). Rotenone, oligomycin, FCCP, antimycin A, menadione, and tetraphenylphosphonium chloride was purchased from Sigma-Aldrich. All other chemicals used for this work were purchased from commercial sources at analytical grade.

### 4.2 Animals

Both young (4–6 months) and old (24–28 months) male F344 rats were acquired from the National Institute on Aging animal colonies. The rats were housed at the Linus Pauling Institute animal facility at Oregon State University and allowed to acclimatize for at least 1 week prior to any experimentation. Animals were maintained on a 12 h light cycle (7am to 7pm) and fed standard chow *ad libitum*. All animal work was approved and in accordance to IACUC guidelines. The AAALAC-accredited Laboratory Animal Resources Center (LARC) provided management and veterinary care. (PHS Animal Welfare Assurance Number D16-00145).

### 4.3 Hepatocyte isolation

Hepatocyte isolation was performed as described previously [67]. Briefly, after anesthesia via AALAC-approved protocols, the liver was perfused via a cannula in the portal vein with Hank's balanced salt solution, pH 7.4. Following removal of blood, liver cells were disassociated using collagenase solution (1 mg/ml). The resultant cell suspension was filtered through sterile gauze to remove connective tissue and debris. Parenchymal cells were isolated using gravity filtration and washed three times with Krebs–Henseleit solution, pH 7.4, bubbled for 30 min with carbogen gas. Cells were resuspended in Kreb-Henseleit solution and placed in a round bottom flask and rotated at room temperature for 1 h before cell count and viability were assessed using trypan blue exclusion.

### 4.4 Analysis of hepatocyte mitochondrial O_2_ consumption

O_2_ consumption rates of young and old rat hepatocytes were determined using an Oxygraph-2k High Resolution Respirometer (Oroboros^®^, Austria) at 25 °C. Young and old rat hepatocytes were treated with either vehicle (0.05% dimethylformamide) or 300 μM menadione for 15 min while being rotated on a MACSMIX (Miltenyi Biotec) rotator. Cells were then pelleted by centrifugation at 100×*g*, washed in Krebs-Henseleit solution to remove excess menadione and set the treatment time. Cells were then pelleted again, the Krebs-Henseleit was removed and cells were resuspended in MiR05 medium to a cellular density of 2.5 × 10^5^ cells/mL before being added to the instrument chamber. Following assessment of basal O_2_ consumption, proton leak-linked O_2_ consumption (Leak) was measured after oligomycin injection (2.5 μM final concentration). Maximal respiratory capacity under uncoupling conditions was measured by successive 0.05 μM additions of the uncoupler FCCP (0.1 μM final concentration was sufficient to fully uncouple respiration in all experiments). In other experiments, electron flux through various portions of the electron transport chain was determined. CI O_2_ consumption was measured by addition of 10 mM glutamate, 2 mM malate, and 2 mM ADP. CI and CII O_2_ consumption were then measured in conjunction by addition of 5 mM succinate. Further differentiation of CI and CII O_2_ consumption was ascertained by addition of 0.5 μM rotenone. Electron transport chain coupling and Leak were then determined by addition of 2.5 μM oligomycin (final concentration). Non-mitochondrial respiration, which was used as a correction factor for O_2_ consumption, was measured after addition of 2.5 μM antimycin A (final concentration).

### 4.5 Digitonin fractionation

In order to allow direct mitochondrial examination by controlling substrate provision while avoiding issues of mitochondrial isolation such as yield, quality, and subpopulation selection, we chose to use digitonin permeabilized primary hepatocytes as first described by Andersson and Jones [68], and later refined by Hagen et al. [69].

Appropriate digitonin concentrations for hepatocytes isolated from young and old rats were determined empirically based on successful permeabilization of the plasma membrane while mitochondrial membranes remained intact. Briefly, hepatocytes were treated with a digitonin concentration gradient (0.01–1.0 mg/10^6^ cells) over a time course (1–10 min). Permeabilization of the plasma membrane was confirmed via lack of trypan blue exclusion (>90% of cells are blue). Cells were then pelleted and glutamate dehydrogenase (GDH) activity was measured in the supernatant and pellet fractions. Mitochondria were considered to be intact and functional when >90% of GDH activity was in the pellet and the mitochondrial respiratory control ratio (RCR), a useful general measure of mitochondrial coupling and function, was above 5. Only preparations with these characteristics were used in experiments. Concentrations of digitonin that met these criteria varied slightly between young and old (0.1 and 0.08 mg/10^6^ cells respectively). Time of treatment had little effect on plasma membrane permeabilization and only affected mitochondrial membranes at higher concentrations (>0.2 mg/10^6^ cells). This digitonin: cell ratio remained effective over varying cellular concentrations tested (0.25 × 10^6^–1 × 10^7^ cells/mL).

### 4.6 Analysis of mitochondrial membrane potential in permeabilized hepatocytes

Δψ_m_ was determined using an Oxygraph-2k High Resolution Respirometer at 25 °C and a potential-sensitive TPP electrode. Permeabilized cells in MiR05 medium were added to the chamber and State I, or substrate-starved O_2_ consumption was ascertained. State IV respiration and Δψ_m_ were then measured upon the addition of 10 mM glutamate, and 2 mM malate in the absence of ADP. State III respiration and Δψ_m_ were determined by addition of 2 mM ADP. Menadione is known to induce redox cycling and increased O_2_ consumption was apparent in these experiments as menadione remained in the chamber for the full time course. As this experiment was determining changes in Δψ_m_ over time, the increased O_2_ consumption did not affect our measures. A standard curve using TPP was created for each experiment by adding tetraphenylphosphonium chloride at concentrations of 0.5, 1.0, 1.5, 2, 2.5, and 3.0 μM prior to the addition of permeabilized hepatocytes to the chamber.

### 4.7 Glutathione analysis

Glutathione (GSH) content of permeabilized cells was determined according to the methods of Jones et al. [70] and modified by Suh et al. [44]. Briefly, suspensions were homogenized in an equal volume of 10% (w/v) perchloric acid (PCA) containing 10 mM EDTA. After deproteinization, 200 μL of the supernatant containing internal standard (γ-glutamyl glutamate) was mixed with 50 μL of 100 mM iodoacetic acid and the pH was adjusted to 9.0 ± 0.2 by using KOH-K_2_B_4_O_7_ buffer (1 M KOH: 1.6 M K_2_B_4_O_7_). Samples were placed in the dark, at room temperature for 1 h. The resulting *S*-carboxymethyl derivatives were subjected to further chemical modification by addition of an equal volume of dansyl-chloride solution (20 mg/mL in acetone). The solution was incubated overnight in the dark and at room temperature. The next day, 500 μL of chloroform was added, samples were vortexed and then allowed to incubate for 1 h in the dark, at room temperature. Samples were then centrifuged at 15,000×*g* for 15 min at 4 °C before removing and assaying the supernatants. Samples (20 μL) were separated by HPLC using a Thermo Scientific (Eugene, OR) APS-2 Hypersil column and detected on a Shimadzu (Kyoto, Japan) SPD-10AVP fluorometric detector set at 335 nm for excitation and 515 nm emission. Quantitation was obtained by integration relative to GSH and GSSG standards.

### 4.8 Statistical analysis

All statistical analyses were performed using Excel (Microsoft, Inc.) and Prism 6 (GraphPad Software, Inc.). Data are represented as mean ± standard error of the mean (SEM). For comparisons between two samples, two-sided Student's t-test was used. Where comparing the means of two or more independent groups with a single independent variable (i.e. between age groups), one-way ANOVAs with Sidak post-hoc analysis for multiple comparisons were used. Where comparing the mean differences between groups with two independent variables (i.e. between age groups and over a time-course), two-way ANOVAs with Tukey's post-hoc analysis for multiple comparisons were used. For two-way ANOVAs, statistical significance between age groups is indicated on the legend of the figures with an asterisk (*), and statistical significance over a time-course (versus t_0_) is indicated by a pound sign (#) over each data point in the figure. Differences between samples that resulted in a p-value of ≤0.05 were considered statistically significant.

## Author contributions

NOT performed most of the experiments, and largely contributed to experimental design and writing of the manuscript. KPS performed part of the experiments and largely contributed to writing of the manuscript. TMH was responsible for experimental design and largely contributed to writing of the manuscript.

## Conflicts of interest

The authors declare that they have no conflicts of interest.
